# An unusual cause of altered airway pressure during surgery under general anaesthesia

**DOI:** 10.4103/0019-5049.72653

**Published:** 2010

**Authors:** Surya K Dube, Sachidanand J Bharati, Hemanshu Prabhakar

**Affiliations:** Department of Neuroanesthesiology, All India Institute of Medical Sciences, New Delhi, - 110 029, India

**Figure 1:**
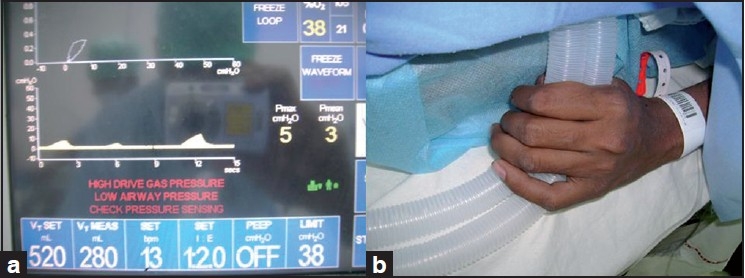
(a) Altered airway pressure alarms (b) Squeezed breathing tubes in the patient’s hand

Sir,

A 17-year-old male, weighing 50 kg, of American Society of Anesthesiologists physical grade I, was scheduled for exploration and repair of right panbrachial plexus injury under general anaesthesia (GA). Inside the operation theatre, we connected routine monitors to the patient. GA was induced with propofol 100 mg and fentanyl 100 mcg. Patient’s airway was secured with a size 3 proseal Laryngeal Mask Airway (LMA). Anaesthesia was maintained with infusion of propofol 100 mcg/kg/min and fentanyl 1 mcg/kg/ hr using a single intravenous cannula of 18G on the dorsum of left hand, along with nitrous oxide in oxygen (2:1 ratio). As the surgery needed intraoperative use of nerve stimulator, use of muscle relaxant was avoided. Mechanical ventilation was adjusted to maintain an end-tidal carbon dioxide (EtCO_2_) of 35–37 mmHg. After 50 minutes of stable proceeding, we noticed a sudden drop in maximum and mean airway pressure along with a ventilator alarm of “high drive gas pressure” [Figure 1a], but the EtCO_2_ reading remained unaltered. We tried looking for any leak or obstruction in the breathing system, inadequate fresh gas flow, displaced LMA, the position of the bag/ventilator selector valve, proper connections and functioning of the ventilator. When all possibilities were ruled out, we noticed that the breathing tubes were squeezed by the patient’s hand [Figure 1b]. Immediately, a bolus of 40 mg of propofol was administered. The patient’s grip over the breathing tube loosened and the airway pressures returned to normal. Our search for the cause revealed that there was an obstruction in the extension line of the propofol and fentanyl infusion was blocked. At the same time, the infusion pump failed to give alarm. The obstruction was cleared and rest of the surgery went uneventful.

High or low airway pressure conditions in the breathing system have been a major cause of anaesthesia related mortality and morbidity.[1] Conditions that can cause low airway pressure alarm include: a disconnection or major leak in the breathing system; inadequate fresh gas flow; leaking tracheal tube cuff; extubation; the bag/ventilator selector valve in bag position; faulty or unconnected ventilator; gas or power supply failure to the ventilator or obstruction upstream to the pressure sensor.[2] In our situation, EtCO_2_ reading was unaltered in spite of altered airway pressure In the presence of dangerously abnormal airway pressures the exhaled carbon dioxide may remain relatively normal.[1] The airway pressure alarms detect the high or low air way pressure conditions in the breathing system. In our case, because of the airway pressure alarm, the obstruction of the propofol and fentanyl infusion was timely detected and subsequent events of inadequate anaesthetic depth could be avoided. We suggest a careful observation of airway pressure alarms in the intraoperative period where we avoid the use of muscle relaxants.
